# The impact of inpatient suicide on psychiatric nurses and their need for support

**DOI:** 10.1186/1471-244X-11-38

**Published:** 2011-03-08

**Authors:** Chizuko Takahashi, Fuminori Chida, Hikaru Nakamura, Hiroshi Akasaka, Junko Yagi, Atsuhiko Koeda, Eri Takusari, Kotaro Otsuka, Akio Sakai

**Affiliations:** 1 Social Support Center Morioka, JT Honcho Bldg. 3F, 1-9-14 Honcho-Dori, Morioka, 020-0015 Japan; 2 Department of Psychiatry, Iwate Medical University, 19-1 Uchimaru, Morioka, 020-8505 Japan; 3 Hospital Management Section of Iwate Medical University Hospital, 19-1 Uchimaru, Morioka, 020-8505 Japan

## Abstract

**Background:**

The nurses working in psychiatric hospitals and wards are prone to encounter completed suicides. The research was conducted to examine post-suicide stress in nurses and the availability of suicide-related mental health care services and education.

**Methods:**

Experiences with inpatient suicide were investigated using an anonymous, self-reported questionnaire, which was, along with the Impact of Event Scale-Revised, administered to 531 psychiatric nurses.

**Results:**

The rate of nurses who had encountered patient suicide was 55.0%. The mean Impact of Event Scale-Revised (IES-R) score was 11.4. The proportion of respondents at a high risk (≥ 25 on the 88-point IES-R score) for post-traumatic stress disorder (PTSD) was 13.7%. However, only 15.8% of respondents indicated that they had access to post-suicide mental health care programmes. The survey also revealed a low rate of nurses who reported attending in-hospital seminars on suicide prevention or mental health care for nurses (26.4% and 12.8%, respectively).

**Conclusions:**

These results indicated that nurses exposed to inpatient suicide suffer significant mental distress. However, the low availability of systematic post-suicide mental health care programmes for such nurses and the lack of suicide-related education initiatives and mental health care for nurses are problematic. The situation is likely related to the fact that there are no formal systems in place for identifying and evaluating the psychological effects of patient suicide in nurses and to the pressures stemming from the public perception of nurses as suppliers rather than recipients of health care.

## Background

Psychiatric disorders have been identified as among the strongest risk factors for suicide [1,2]. Psychiatric inpatients thus constitute a high-risk group for suicide attempts. For these reasons, nurses working in psychiatric hospitals and psychiatric wards are more prone to encounter suicidal ideation in patients and attempted or completed suicides than nurses in other departments.

Previous studies have reported that patient suicide have a severe emotional impact in some psychiatrists and psychiatric trainees [3], and personal grief in therapists [4]. The completed suicide of a patient represents a critical event for a nurse who was in charge of or had some contact with the patient, and the nurse may blame him/herself and experience feelings of worthlessness associated with his/her inability to prevent the patient's death [5,6]. However, results from one study show that only 34% of 106 surveyed psychiatric hospitals throughout Japan provided mental health care programmes for their staff following patient suicide [7]. Current Japanese psychiatric care systems have been slow to adopt staff-oriented programmes on suicide and post-suicide care, partly due to persistent resistance and a distorted view of suicide on the part of psychiatric health care providers [8].

This study investigated the experiences of psychiatric nursing staff exposed to patient suicide, self-evaluation of post-suicide stress, and availability of suicide-related mental health care services and education. The purpose of this study was to investigate issues related to patient suicide in mental health nursing in the hopes of contributing to the mental health of nurses.

## Methods

A survey questionnaire, together with the IES-R, was sent over a period from July 18, 2008 to August 1, 2008 to 562 psychiatric nurses working at eight psychiatric medical institutions (two general and six psychiatric hospitals) located in City A and its environs in the northern Tohoku region of Japan. The nurses surveyed worked in inpatient wards, outpatient clinics, day-care services, and other related facilities. All of the respondents had experience working in psychiatric inpatient wards.

The questionnaire was designed and prepared for this study based on suicide-related papers previously published in Japan[9-11], documents related to the Basic Law on Suicide Countermeasures (2006), survey items used in a survey conducted among the general public by the Cabinet Office (2007), and survey items used by Minami and colleagues[7]. We did not perform any pilot study in advance of the current questionnaire survey, determining it unnecessary, given that our survey consists of questions based on the aforementioned Law, the results of reliable studies, and other reliable information. All survey items were carefully assessed in advance by psychiatrists, public health nurses, and experts in ethics and statistics.

The questionnaire, which is printed on A4 paper and consists of eight pages including the cover page, is divided into the following four major sections: 'Your perception of suicide', 'Patient suicide', 'Current on-site support systems', and 'About yourself'. Each section has roughly ten subsections. In addition, each subsection contains roughly ten questions.

The questionnaire as a whole asked questions on (1) the experience of exposure to completed inpatient suicide, (2) the availability of mental health care services for affected nursing staff and the perceived need for post-event mental health care initiatives, and (3) on-site support systems. The final item (3) concerned the presence and scope of educational training conducted for the professional development of psychiatric nurses. More specifically, questions involved such matters as whether on-site training had been conducted in the preceding three years relating to stress-coping methods, suicide prevention measures, mental health care for nurses, risk management, etc., and whether the respondents ask questions or whether they have anyone to consult with when they encounter an unfamiliar problem at work. The purpose of asking this series of questions was to determine the level of opportunity available to them to develop a better understanding of suicide and suicide prevention, and how much training there is on suicide-related issues, such as psychiatric disorders and stress. In addition, nurses who witnessed a patient suicide attempt were asked about the situation at the time of the encounter, the degree of relationship with the patient, and related questions.

The IES-R sent with the questionnaire is a post-traumatic stress symptom scale. It is designed to measure, with its Intrusion, Hyperarousal, and Avoidance subscales, the three types of PTSD symptoms that are said to occur after traumatic experiences such as crime, disasters, and accidents. The validity and reliability of the scale have been demonstrated by others[12].

Sufficient care was taken to maintain confidentiality; individuals who completed the questionnaire remained completely anonymous. The questionnaire sheets were placed in envelopes and distributed to each respondent individually. The respondents themselves sealed their responses in the envelopes before submission. Consent to the survey was assumed on the part of respondents who opted to complete and return the form. This study was approved by the Iwate Medical University Ethics Committee.

Statistical analysis (chi-square) was performed using the IBM SPSS Statistics software package (Version 16J, SPSS Japan Inc., Tokyo, Japan).

## Results

A total of 531 individuals (94.5%) responded (Table 1). This high response rate was probably due to the prior explanations of the gist of this study and the requests for cooperation put in to the management of the medical institutions (directors of hospitals and managers of nursing departments) verbally and in writing.

**Table 1:** Respondent characteristics

		Total		Females		Males		No/inappropriate answer	
		n = 531		n = 332		n = 153		n=46	
		n	%	n	%	n	%	n	%
Mean age, +/- SD		41.9	± 12.3	43.6	± 12.4	38.2	± 11.1		
	20 to 29 years	91	17.1	56	16.9	35	22.9	0	0.0
	30 to 39 years	99	18.6	50	15.1	49	32.0	0	0.0
Age group	40 to 49 years	135	25.4	97	29.2	38	24.8	0	0.0
	50 to 59 years	97	18.3	82	24.7	15	9.8	0	0.0
	60 years or older	36	6.8	29	8.7	7	4.6	0	0.0
	No/inappropriate answer	73	13.7	18	5.4	9	5.9	46	100.0
Total years of nursing experience, +/- SD		17.9	± 11.9	19.8	± 12.0	14.0	± 10.8		
	Maximum years	55.0		55.0		47.0			
	Minimum years	0.1		0.1		0.3			
	No/inappropriate answer	65	12.2	14	4.2	5	3.3	46	
Total years as psychiatric nurse, +/- SD		11.8	± 1.0	11.4	± 9.0	12.6	± 10.6		
	Maximum years	47.0		36.3		47.0			
	Minimum years	0.1		0.1		0.2			
	No/inappropriate answer	61	11.5	11	3.3	5	3.3	46	
	Registered nurse	317	59.7	209	63.0	108	70.6	0	0.0
Distinction	Licensed practical nurse	155	29.2	113	34.0	42	27.5	1	2.2
	No/inappropriate answer	59	11.1	10	3.0	3	2.0	45	97.8
	Management: chief nurse or higher	61	11.5	36	10.8	25	16.3	0	0.0
Post	Other	391	73.6	273	82.2	117	76.5	0	0.0
	No/inappropriate answer	79	14.9	23	6.9	11	7.2	46	100.0

Description: Background factors of the subjects,i.e.,mean age,total years of nursing experience,total years of psychiatric nursing experience, distinction of nursing staff, ,post,experience of suicide someone close.

The female/male ratio of the respondents was greater than 2:1. The overall average age was early forties; the mean age was higher for female respondents than male. The mean duration of psychiatric nursing experience was 11.8 ± 1.0 years out of 17.9 ± 11.9 years' professional nursing experience. About 60% (n = 317) of the respondents were registered nurses, while some 30% (n = 155) were licensed practical nurses. Nurses in Japan are classified as either "registered nurses", who have passed a national examination and are granted a license by the Minister of Health, Labour, and Welfare, or "licensed practical nurses", who are granted a license by the governor of one of the 47 prefectures to perform nursing services under the direction of registered nurses. Eleven and a half per cent (n = 61) of them held management-level positions (chief nurse or higher), and more than 70% (n = 391) engaged in general nursing services.

### 1. Experience with completed inpatient suicide (Table 2)

**Table 2:** Rates of nurses who encountered completed patient suicide and details of the encounter.

		No.of respondents(%)
Did you encounter an inpatient suicide?	Yes	292 (55.0)
(n = 531)	No	209 (39.4)
	No/inapporopriate answer	30 (5.6)

How many years have passed since the encounter?	20 or more years	15 (5.1)
(n = 292)	10 to 20 years	55 (18.8)
	5 to 10 years	29 (9.9)
	3 to 5 years	51 (17.5)
	1 to 2 years	84 (28.8)
	No/inapporopriate answer	58 (19.9)

In which time period?	0 a.m. to 7 a.m.	70 (24.0)
(n = 292)	8 a.m. to 4 p.m.	40 (13.7)
	5 p.m. to 11 p.m.	45 (15.4)
	No/inapporopriate answer	137 (46.9)

Where did it take place?	In the psychiatric ward	162 (55.5)
(n = 292)	Outside the hospital (in the patient's home or during an outing)	102 (34.9)
	Other	14 (4.8)
	No/inapporopriate answer	14 (4.8)

When the event happened,you were:	Off from work	75 (25.7)
(n = 292)	Prior to going off duty	33 (11.3)
	On duty	58 (19.9)
	On duty and involved in post-event treatment	39 (13.4)
	Out of hospital	83 (28.4)
	No/inapporopriate answer	4 (1.4)

How closely were you involved with the patient?	No involved	26 (8.9)
(n = 292)	Little involvement	64 (21.9)
	Not charged but involved	187 (64.0)
	Charged	10 (3.4)
	No/inapporopriate answer	5 (1.7)

Description: Data of the subjects,i.e., the fact that nurses encountered patients' suicide, years since the encounter, time and place of the encounter, situation when the encounter took place, involvement and relation of nurses to suicidei

More than half of the respondents (n = 292) stated that they had experienced a case of completed suicide by an inpatient. Of those, 28.8% of the experiences (n = 84) occurred comparatively recently, within one or two years. One-fourth of the nurses (n = 70) encountered suicides from midnight to early morning hours. More than half of the suicide attempts (n = 162) occurred in the psychiatric ward. More than 60% of the nurses (n = 191) were not in physical proximity to the patient at the time of the suicide. The following situations were typical: the patient was given overnight home leave or permission to go on an outing, the nurse was off duty, or the nurse had yet to leaving home for work. However, more than 30% of the reported cases (n = 97) took place during working hours, and in several cases the nurse was involved in attempts to resuscitate the patient. Although only a small proportion of respondents (n = 10) answered that they were the designated nurse in charge of the patient who completed suicide, more than 60% of the surveyed nurses (n = 187) indicated that they had at least some contact with the patient.

Although not shown in the table, the number of nurses who encountered patient suicide during their working hours and were involved in the transport of the deceased or similar procedures was 39 (13.4%).

### 2. IES-R scale evaluation

The severity of PTSD symptoms was measured using the IES-R scale for 292 nurses who had been exposed to inpatient suicide events to evaluate the psychological impact of the incident (Table 3). The PTSD high-risk group was defined as those scoring 25 or higher on the 88-point IES-R scale, based on the screening results reported by Asukai [9]. The number of PTSD high-risk individuals was 40 (13.7%).

**Table 3:** IES-R score for nurses who had experienced inpatient suicide.

	Not at all		A little bit		Moderately		Quite a bit		Extremely		No/inappropriate answer		Total	
	n	%	n	%	n	%	n	%	n	%	n	%	n	%
Any reminder brought back feelings about it	63	21.6	117	40.1	63	21.6	25	8.6	15	5.1	9	3.1	292	100
I had trouble staying asleep	206	70.5	37	12.7	28	9.6	8	2.7	1	0.3	12	4.1	292	100
Other things kept making me think about it	189	64.7	58	19.9	24	8.2	6	2.1	6	2.1	9	3.1	292	100
I felt irritable and angry	234	80.1	38	13.0	7	2.4	4	1.4	0	0.0	9	3.1	292	100
I avoided letting myself get upset when I thought about it or was reminded of it	145	49.7	91	31.2	24	8.2	17	5.8	5	1.7	10	3.4	292	100
I thought about it when I didn't mean to	133	45.5	100	34.2	31	10.6	14	4.8	3	1.0	11	3.8	292	100
I felt as if it hadn't happened or wasn't real	199	68.2	46	15.8	21	7.2	5	1.7	1	0.3	20	6.8	292	100
I stayed away from reminders about it	197	67.5	53	18.2	15	5.1	7	2.4	3	1.0	17	5.8	292	100
Pictures about it popped into my mind	193	66.1	59	20.2	14	4.8	8	2.7	3	1.0	15	5.1	292	100
I was jumpy and easily startled	172	58.9	63	21.6	24	8.2	11	3.8	9	3.1	13	4.5	292	100
I tried not to think about it	152	52.1	67	22.9	34	11.6	18	6.2	7	2.4	14	4.8	292	100
I was aware that I still had a lot of feelings about it,but I didn't deal with them	165	56.5	62	21.2	26	8.9	17	5.8	6	2.1	16	5.5	292	100
My feelings about it were kind of numb	162	55.5	70	24.0	35	12.0	6	2.1	2	0.7	17	5.8	292	100
I found myself acting or feeling as though I was back at that time	214	73.3	42	14.4	17	5.8	4	1.4	1	0.3	14	4.8	292	100
I had trouble falling asieep	212	72.6	38	13.0	14	4.8	10	3.4	4	1.4	14	4.8	292	100
I had waves of strong feelings about it	192	65.8	53	18.2	22	7.5	9	3.1	5	1.7	11	3.8	292	100
I tried to remove it from my memory	201	68.8	42	14.4	21	7.2	9	3.1	4	1.4	15	5.1	292	100
I had trouble concentrating	226	77.4	40	13.7	9	3.1	3	1.0	1	0.3	13	4.5	292	100
Reminders of it caused me to have physical reactions,such as sweating,trouble breathing,nausea,or a pounding heart	227	77.7	38	13.0	12	4.1	4	1.4	0	0.0	11	3.8	292	100
I had dreams about it	246	84.2	28	9.6	4	1.4	1	0.3	0	0.0	13	4.5	292	100
I felt watchful or on-guard	112	38.4	92	31.5	41	14.0	20	6.8	14	4.8	13	4.5	292	100
I tried not to talk about it	187	64.0	54	18.5	22	7.5	11	3.8	5	1.7	13	4.5	292	100

Description: Scores of IES-R (Impact of Event Scale-Revised [Weiss & Marmar, 1997]).

Each question was rated on a five-point scale. Particularly strong responses were noted for: two intrusion-related symptoms, i.e. #1 ("Any reminder brings back feelings about it") and # 6; three avoidance-related symptoms, i.e. #5 and #11 ("I tried not to think about it") and #12); and one hypervigilance-related symptom, namely #21 (I felt watchful or on-guard).

### 3. Availability of mental health care programmes and the perceived need for them

About 80% (n = 234) of the nurses who encountered inpatient suicide responded that no mental health care programmes were implemented for nursing staff following the suicide (Table 4). Our questionnaire included statements on whether or not their hospitals provided occasions for suicide-related discussion or a case study review after the event. Those questions were adopted by revision and supplementation of the survey items adopted by Minami [7].

**Table 4:** Post-suicide follow-up care programs for nurses.

		No.of respondents(%)
Mental health care program	Implemented	46(15.8)
(n = 292)	Not implemented	234(80.1)
	No/inapporopriate answer	12(4.1)

Case study session was:	Held at safety committee	23(7.9)
(n = 292)	Held at ward/unit meeting	68(23.3)
	Held by limited staff	75(25.7)
	Other	24(8.2)
	Not held	83(28.4)
	No/inapporopriate answer	19(6.5)

Description: Whether mental health care programs and case study sessions were available for nurses who encountered patients' suicide.

Most of the responses favoured implementing staff-oriented mental health care programmes following a suicide, and recognized the need for developing a more compassionate attitude toward suicide, as well as the need for sharing information on the course of events leading up to the suicide (Table 5).

**Table 5:** Questions concerning need for mental health care programs.

	Not at all		Don't think so		Think so		Strongly think so		No/inappropriate answer		Total	
	n	%	n	%	n	%	n	%	n	%	n	%
Mental health care programs following patient suicide are necessary for medical staff.	1	0.2	22	4.1	153	28.8	283	53.3	72	13.6	531	100.0
Mental health issues cannot be solved by oneself.	4	0.8	57	10.7	315	59.3	122	23.0	33	6.2	531	100.0
Inaction worsens mental health issues.	7	1.3	60	11.3	323	60.8	104	19.6	37	7.0	531	100.0
Inappropriate mental health care may cause staff to resign.	9	1.7	90	16.9	259	48.8	138	26.0	35	6.6	531	100.0
Mental health care programs help to prevent suicide.	12	2.3	97	18.3	290	54.6	94	17.7	38	7.2	531	100.0
Proper knowledge about suicide should be needed.	2	0.4	18	3.4	276	52.0	201	37.9	34	6.4	531	100.0
Staff should share information on the course of events that led to suicide.	1	0.2	25	4.7	268	50.5	204	38.4	33	6.2	531	100.0
Mental health care programs should cover all medical staff.	5	0.9	43	8.1	278	52.4	169	31.8	36	6.8	531	100.0
Mental health care programs will reduce staff's psychological burdens.	5	0.9	61	11.5	305	57.4	124	23.4	36	6.8	531	100.0
Discussion about the cause of suicide is useful for mental health management.	6	1.1	81	15.3	304	57.3	103	19.4	37	7.0	531	100.0
Mental health care programs involving external professionals should be considered.	7	1.3	99	18.6	290	54.6	98	18.5	37	7.0	531	100.0

Description: Whether the nurses recognized the need for mental health programs.

A significant difference was observed in the amount of knowledge and awareness regarding suicide and in the perceived need for mental health care for staff members between nurses in managerial positions and other nurses. However, no significant difference was observed between licensed practical nurses and registered nurses.

### 4. On-site support systems

When respondents were asked about the topics of on-site seminars held in the previous three years, more than 50% of the answers reported seminars or workshops on 'psychiatric diseases' (60.3%, n = 320) and 'risk management' (54.4%, n = 289). However, less than 20% indicated 'group approaches' (11.1%, n = 59), 'mental health care for nursing staff' (12.8%, n = 68), and 'stress-coping methods' (16.9%, n = 90). Rates of those who had attended a 'suicide and suicide prevention measures' seminar were higher, with a response of 26.4% (n = 140) (Table 6).

**Table 6:** Subjects of on-site seminars held in the previous three years.

	No		Yes		No/inappropriate answer		Total	
	n	%	n	%	n	%	n	%
Stress-coping methods	345	65.0	90	16.9	96	18.1	531	100.0
Suicide and suicide prevention measures	301	56.7	140	26.4	90	16.9	531	100.0
Psychiatric diseases	139	26.2	320	60.3	72	13.6	531	100.0
Risk management	161	30.3	289	54.4	81	15.3	531	100.0
Mental health care for nursing staff	367	69.1	68	12.8	96	18.1	531	100.0
Skill upgrade and career advancement	258	48.6	171	32.2	102	19.2	531	100.0
Cooperation with other professions	289	54.4	145	27.3	97	18.3	531	100.0
Group approaches	367	69.1	59	11.1	105	19.8	531	100.0

Description: Variety of themes of on-site seminars for nurses.

## Discussion

Although this study focused only on cases of completed suicide, more than half (55.0%) of respondents had had experience with patient suicide. Previous studies on psychiatric nurses exposed to patient suicide reported rates of 58.3% [10] and 32.4% [11]. Another study indicated that 66% of psychiatric hospitals or hospitals with psychiatric wards (the total number is 106) reported suicidal events [7]. These previous reports, however, include unsuccessful suicide attempts. In this regard, a very high rate was obtained from the current study. In Japan, the annual number of suicides has exceeded thirty thousand for 12 consecutive years, and the suicide rate is exceptionally high compared to the figures in other advanced countries. The suicide rate in the prefecture, where respondents to the current questionnaire survey are located, is one of the highest in Japan[13]. The present survey results may reflect this regional tendency.

More than half of the nurses who encountered suicide by patients stated that they had had at least some contact with them. Patient suicide is an extremely serious incident for medical professionals. The few studies published suggest that it is quite common for residents to encounter patient suicide during their training and that they undergo significant levels of psychological stress [14]. Sudak suggested that the feelings experienced by residents and clinicians following the suicide by patient are quantitatively smaller than in the case of suicide by a family member but are similar to them qualitatively [15].

Despite some difficulties in comparison arising from the use of different methods and other factors, similar IES-R scores have been reported for significantly disastrous events. In a study evaluating general traumatic events (e.g., physical abuse, sexual harassment, obsessive relational intrusion, becoming the target of unwanted romantic attention, and patient suicide) among 124 psychiatric nurses, the mean IES-R score was 13.4, and 18 nurses (14.5%) were classified in the high-risk group [16]. The mean IES-R subscale scores (avoidance, intrusion, hypervigilance) obtained in this study were compared with those in a preceding study [16].

In this study, most of the nurses indicated the desire for mental health care programmes for health care workers who have experienced a shocking event on the ward. This indicates that most psychiatric nurses are aware of the need for staff-oriented mental health care services. In actual terms, however, only 15.8% of the respondents reported the availability of mental health care programmes for health care workers following a suicide event. In Japanese medical practice, it is often the case that when a patient completes suicide, the situation is not conducive to the provision of psychological assistance for nurses in charge of the patient [17]. The results obtained in this study reflect in part current style of Japanese psychiatric practice management, as shown above.

Nonetheless, inadequacies at facilities overseas have been reported as well. For example, Mangurian and colleagues reported that, when they encountered patient suicide during their own residencies, they found that emotional support and support by medical institutions were lacking [18].

The patient suicide-related issues considered here derive from both the circumstances of the profession and nurses' perception of their own social role. Expressing bitter feelings is often considered by nurses and other medical professionals to be giving in to one's weaknesses and exposing one's helplessness to others. Nurses fear that disclosure of their weaknesses would damage their professional reputation, and this fear could be one of the reasons for not speaking up.

Consequently, nurses who have lost a patient due to suicide are troubled by the thought that they may be responsible for the death. This sense of guilt and self-condemnation can result in depression and other PTSD symptoms and can affect professional identity and nursing skills and duties [6]. In addition, other nurses may develop a fear of going through a similar event again [19], which can lead to a dysfunctional medical care system.

For these reasons, the timely implementation of appropriate mental health care programmes for nursing staff who have been through a patient suicide is a significant part of creating an effective medical care environment. Providing staff-oriented post-suicide mental health care programmes falls under the category of postvention activities. Postvention activities should enable verbal expression of the emotional shock of the bereaved. They also serve to liberate staff from the vicious circle of the depression that may result from the mistaken belief that the affliction is their own and no one else's. Moreover, they help staff members to unite through mutual support [5]. Group meetings can help nurses realize 'the universality of grief and reduce self-blame and excess responsibility' [20]. The daily practice of examining and sharing one's feelings paves the way for sharing deep emotional feelings associated with a patient's suicide [21]. This is not an issue exclusively for nurses. Sudak points out the importance of sharing opinions on suicide and freely discussing concerns with other residents [22]. In addition, Balon discusses the significance of the impact of patient suicide, looking back at an experience which he himself had during his residency, and asserts the usefulness of psychological autopsies shared with others for metal health care [23].

Among other interventions, nurses need suicide prevention education. As revealed by the present study, however, only a very low proportion of medical institutions provide on-site suicide-related seminars. Suicide-related issues may be dealt with at seminars on risk management, which were held frequently according to our results. Risk management approaches can be applied to intervention and the prevention of inpatient suicide. Patients' risk factors for suicide are evaluated from information collected from admissions, and therapeutic and nursing plans are developed based on the results of the evaluation. Implementation of these plans helps identify subtle changes in behaviour. Such systems involve patients, their families, nurses, physicians, and other health care workers in the risk management programmes for suicide prevention [6].

Practical examples of suicide prevention education for medical and nursing students and nurses have been reported [24,25]. However, a majority of these programmes have been tailored to those engaged in prevention; relatively few programmes have provided information and support based on the assumption that the attendees might be affected by a patient's suicide. Suicide prevention education is invaluable. Nurses should be aware that they may be forced to deal with the suicide of patients or persons close to them.

This study revealed that in the medical institutions targeted for the survey, there is a lack of awareness of the impact of completed patient suicide in nurses, and that the need to educate nurses on issues related to suicide has not gained wide recognition. These conclusions are expected to apply to many different regions throughout Japan. Accordingly, we hope that this study will provide grounds for improving the present situation.

## Limitations

We must be very careful about generalizing our findings to any nurse whose patient completes suicide, for the evidence as provided here comes from Japanese settings. Moreover, this study focused on psychiatric nurses in urban areas; nurses in other geographical areas and other medical professions were not addressed.

Though the questionnaire used in this study was based on the extant literature, including suicide-related papers, documents related to the Basic Law on Suicide Countermeasures and others, its validity and reliability has not yet been tested. A pilot study is therefore necessary if the questionnaire is to be put to use in the future.

It is generally reported that female nurses are at a higher risk for PTSD and related conditions than male nurses. However, gender differences in IES-R scores were not taken into consideration in this study.

## Conclusions

The current study revealed that more than half of respondents (nurses) had experienced a patient's completed suicide. The IES-R score obtained for these nurses suggest that inpatient suicide was a significantly distressing event. However, though most of these nurses were aware of the need for staff-oriented mental health care services, systematic post-suicide mental health care programmes for nurses are rarely available. Moreover, initiatives for education on issues related to suicide and mental health care are lacking. Hence, improvement in support systems is needed to provide effective mental health care for nurses so that they can work more effective at primary prevention.

This study may be useful in providing grounds to improve the present situation by highlighting the significance of the impact of completed patient suicide on nurses, and the lack of mental health care and the ill-prepared educational system regarding issues related to patient suicide.

## Competing interests

The authors declare that they have no competing interests.

## Authors' contributions

CT and FC conceived of the study and participated in its design and coordination. KO and AS helped to draft the manuscript. HN performed the statistical analysis. HA, JY, AK and ET have made contributions to acquisition and analysis of data. All authors read and approved the final manuscript.

## Pre-publication history

The pre-publication history for this paper can be accessed here:

http://www.biomedcentral.com/1471-244X/11/38/prepub
